# CURE-Chloroplast: A chloroplast C-to-U RNA editing predictor for seed plants

**DOI:** 10.1186/1471-2105-10-135

**Published:** 2009-05-08

**Authors:** Pufeng Du, Liyan Jia, Yanda Li

**Affiliations:** 1 MOE Key Laboratory of Bioinformatics and Bioinformatics Div. TNLIST/Department of Automation, Tsinghua University, Beijing 100084, PR China

## Abstract

**Background:**

RNA editing is a type of post-transcriptional modification of RNA and belongs to the class of mechanisms that contribute to the complexity of transcriptomes. C-to-U RNA editing is commonly observed in plant mitochondria and chloroplasts. The *in vivo *mechanism of recognizing C-to-U RNA editing sites is still unknown. In recent years, many efforts have been made to computationally predict C-to-U RNA editing sites in the mitochondria of seed plants, but there is still no algorithm available for C-to-U RNA editing site prediction in the chloroplasts of seed plants.

**Results:**

In this paper, we extend our algorithm CURE, which can accurately predict the C-to-U RNA editing sites in mitochondria, to predict C-to-U RNA editing sites in the chloroplasts of seed plants. The algorithm achieves over 80% sensitivity and over 99% specificity. We implement the algorithm as an online service called CURE-Chloroplast .

**Conclusion:**

CURE-Chloroplast is an online service for predicting the C-to-U RNA editing sites in the chloroplasts of seed plants. The online service allows the processing of entire chloroplast genome sequences. Since CURE-Chloroplast performs very well, it could be a helpful tool in the study of C-to-U RNA editing in the chloroplasts of seed plants.

## Background

RNA editing is a kind of RNA processing (like splicing, 5'capping and 3' polyadenylation) that can alter the genetic information of RNA via insertion, deletion or substitution of single or multiple nucleotides. In plant mitochondrial and chloroplast transcripts, several cytidines can be converted to uridines by a deamination process [1-3]. Generally, there are about 300 to 500 C-to-U RNA editing sites in the mitochondrial transcriptomes of seed plants [4-7], but only 30 to 50 can be found in their chloroplasts [8-12]. Most of the known C-to-U RNA editing instances in plant organelles share a similar property: they are non-synonymous and alter the encoded protein sequence to be more conserved across species than the protein sequence predicted from genomic DNA [13-16]. This effect makes the actual protein sequence different from the sequence predicted from the genomic DNA. Thus, knowledge of C-to-U RNA editing in plant organelles is important in order to correctly analyze the protein sequence and gene expression in both computational and experimental studies.

Biologically, the *in vivo *site recognition mechanism of C-to-U RNA editing in plant organelles is still not fully understood [17]. While several short sequences in the upstream regions of the editing sites have been identified as being critical for site recognition [18-22], little is known about the factors that recognize these sites. Recent studies have suggested that the PPR family of proteins is related to site recognition [23-26]. It seems that every single site or every small set of editing sites are recognized by a specific factor [27,28]. With the above biological knowledge as a basis, machine learning algorithms were introduced to predict C-to-U RNA editing sites in mitochondria. Cummings and Myers proposed the first prediction algorithm for C-to-U RNA editing sites in mitochondria based on the classification tree algorithm [29], REGAL introduced the genetic algorithm [30,31] and Prep-Mt [32] and our CURE (Cytidine-to-Uridine Recognizing Editor) algorithm [33] considered the evolutionary information. Yura et al. proposed a method (RNAE) for predicting the C-to-U RNA editing sites in the chloroplasts of one particular moss organism, *Takakia lepidozioides *[34]. However, the application of RNAE on the seed plant chloroplast genes results in very poor accuracy (Additional file 1). In other words, RNAE is designed for only one moss organism but cannot be used as a predictor for seed plant organisms.

Although the chloroplast C-to-U RNA editing sites of rice [35], maize [8], tobacco [10], sugarcane [36], peas [37], orchids [38] and several other seed plants have been systematically determined through experiments, there is still no available software for predicting the chloroplast C-to-U RNA editing sites in seed plants. This may be due to the relatively small number of editing instances in the chloroplast transcriptomes of these organisms, which makes algorithm design and evaluation very difficult.

However, in the public sequence database, there are many chloroplast transcripts with undetermined RNA editing status. An accurate computational prediction method would be helpful in filling the gap between the sequence data and the RNA editing annotations. It would also reduce the need for experimental determinations, which are costly and time consuming. In this paper, we will extend our algorithm CURE, which can accurately predict C-to-U RNA editing sites in mitochondria, to predict C-to-U RNA editing sites in the chloroplasts of seed plants. We will also present the online prediction service, CURE-Chloroplast.

## Implementation

### Dataset

There are three main RNA editing databases: dbRES [39], REDIdb [40] and EdRNA [41]. REDIdb is the only database focusing on organelle RNA editing sites. We collected all the C-to-U RNA editing sites of seed plant chloroplasts in REDIdb. The duplicate annotations in the database were discarded. The inaccurate annotations, which were associated with nucleotides other than cytidine, were corrected according to the original literatures or the GenBank annotations. The editing sites in pea [37], sugarcane [36] and orchid [38] chloroplasts were extracted from the literatures and added into the dataset. The sequences in this dataset were categorized by the gene name. ClustalW was used to create alignments for each gene. These alignments were used to train CURE-Chloroplast. Table 1 shows the summary of our dataset.

**Table 1:** The summary of the dataset

Organism	No. of Genes	Total	POS	NEG
*Arabidopsis thaliana*	13	2284	28	2256
*Atropa belladonna*	14	2885	27	2858
*Nicotiana sylvestris*	17	1960	35	1925
*Nicotiana tabacum*	17	3712	32	3680
*Nicotiana tomentosiformis*	16	1921	33	1888
*Oryza sativa*	10	2362	20	2342
*Phalaenopsis aphrodite*	22	3802	42	3760
*Pinus thunbergii*	13	1658	28	1630
*Pisum sativum*	16	2839	26	2813
*Saccharum officinarum*	13	3311	23	3288
*Zea mays*	13	3294	25	3269
Overall	164	30028	319	29709

The Number of Genes column is the number of edited genes in the organism, the Total column is the number of all cytidines in the edited genes, the POS column is the number of edited cytidines in the edited genes and the NEG column is the number of unedited cytidines in the edited genes.

This dataset has significant lineage bias. Most of the editing sites are from angiosperms. Only one gymnosperm plant is included. Since several editing sites in the chloroplasts of the gymnosperm are not conserved in angiosperms [9], we need to develop different strategies to predict the C-to-U RNA editing sites in angiosperms and gymnosperms.

### The basic CURE-Chloroplast algorithm

The basic algorithm for CURE-Chloroplast is the same as the CURE algorithm, which we have already successfully developed to predict mitochondria C-to-U RNA editing sites [33]. The CURE algorithm was based on the fact that if one editing site can be found in a column of a multiple sequence alignment of homologous genes from different organisms, it is likely to find another in the same column. In the CURE algorithm, we proposed the concept of Evolutionary Potential Editing Sites (EPESs). In a multiple sequence alignment, if a column contains an editing site, this column is defined as an EPES. An EPES is described with three different elements: a flanking consensus sequence, a conservative ratio and a set of sequences that generate the flanking consensus sequence. The training procedure scans the alignments to collect the EPESs and save them in a database. When the editing sites of a sequence need to be predicted, the prediction procedure uses the BLAST program to map the EPES consensus sequences in the database to this sequence and determines the editing status of every cytidine. The details of the basic CURE algorithm can be found either in our previous work [33] or in additional file 2 of the current paper.

The alignments for training can be prepared by two different methods. One is to create alignments from the CDS sequences of each gene with the ClustalW program; the other is to create alignments directly from the whole genome sequences using the TBA program [42]. Since most of the RNA editing sites in chloroplasts are found within the coding regions, we only use the former method to prepare the alignments. However, CURE-Chloroplast users are still allowed to enter nucleotide sequences without any restrictions – even the entire chloroplast genome sequence can be directly entered. In this situation, only those RNA editing sites in gene regions can be identified because our training dataset contains no information from the non-coding regions.

### CURE-Chloroplast algorithm extended for gymnosperms

As we have mentioned in the dataset section, several editing sites in gymnosperms are not conserved to the angiosperms. Thus, we need to modify the CURE algorithm to make it applicable to gymnosperms. An extended definition of an EPES was proposed. If a column in the multiple sequence alignments contains only T, and this column is at the middle position of a codon, this column is also defined as an EPES. This extended EPES definition is based on the fact that the protein translated from the edited version of the transcript is usually more conserved across species than the sequence predicted from the genomic DNA. Thus, if a cytidine is mapped by this extended EPES, it is likely to be edited to restore the conservation at the protein level.

### Evaluation

Six summary statistics were used to describe the performance of CURE-Chloroplast: sensitivity, specificity, accuracy, positive predictive value (PPV), balanced accuracy (BA) and the Matthew's correlation coefficient (MCC) (Eq. 1~Eq. 6).

(1)

(2)

(3)

(4)

(5)

(6)

TP, TN, FP and FN are the number of true positives, true negatives, false positives and false negatives, respectively.

### Online Service

CURE-Chloroplast was implemented on a Linux server with two Xeon dual-core CPUs and 4 GB of memory. Users can either paste a single sequence or upload a FASTA file containing multiple sequences via the web-based user interface. The server-side PHP scripts process the sequences and carry out the prediction. The results are presented in a web-based result browser. The plain text-based result file can be downloaded within 24 hours after the prediction is made.

CURE-Chloroplast users are allowed to adjust several parameters after the advanced mode is enabled. The "Up Bound" and "Low Bound" parameters define the working region of a K-NN classifier, which is called a "micro-analyzer" in the CURE-Chloroplast system. When the submitted sequence is mapped by an EPES with an RNA editing conservative ratio between the Low Bound and Up Bound, the K-NN classifier will be used to decide whether or not the EPES mapping cytidine should be predicted as an editing site. If the users enlarge the working region bounded by these two parameters, the editing statuses of more cytidines are determined by the K-NN classifier rather than the RNA editing conservative ratio. According to our experience, the default parameters are suitable for most organisms.

The CURE-Chloroplast service has two more options than the CURE service. One is the "Cons-T EPES" option. If this option is turned on, the extended EPES definition will be used. The other is the "Positive strand only" option. If this option is turned on, CURE-Chloroplast will only scan the positive strand of the input sequence.

## Results and Discussion

### Prediction performance analysis

We used leave-one-species-out cross-validation to estimate the performance of CURE-Chloroplast. When we were testing the performance on one organism, all the data relating to that organism, including sequences and editing sites, were removed from the training set. The algorithm was retrained on the remaining data. The details of CURE-Chloroplast performance can be found in Table 2. Overall, CURE-Chloroplast achieved over 80% sensitivity and over 99% specificity. Although the negatives were much more than the positives in the dataset, we found that the sensitivity was still acceptable for most species. CURE-Chloroplast can identify the tiny number of positives among the extremely large number of negatives. Because the dataset was significantly unbalanced, we provided the PPV and MCC values as measures of performance on the unbalanced dataset. Since a similar performance estimation problem in Prep-Mt was solved by introducing balanced accuracy statistics [32], we also provided the balanced accuracy values. The balanced accuracy can be considered as the estimation of accuracy on a balanced dataset.

**Table 2:** The performance of leave-one-species-out cross-validation

Organism	Sen	Spe	PPV	ACC	BA	MCC
*Arabidopsis thaliana*	71.43%	99.87%	86.96%	99.52%	85.65%	0.79
*Atropa belladonna*	92.59%	99.79%	80.65%	99.72%	96.19%	0.86
*Nicotiana sylvestris*	91.43%	99.90%	94.12%	99.74%	95.66%	0.93
*Nicotiana tabacum*	90.63%	99.84%	82.86%	99.76%	95.23%	0.87
*Nicotiana tomentosiformis*	90.91%	99.74%	85.71%	99.58%	95.32%	0.88
*Oryza sativa*	100.00%	99.87%	86.96%	99.87%	99.94%	0.93
*Phalaenopsis aphrodite*	40.48%	99.89%	80.95%	99.24%	70.18%	0.57
*Pinus thunbergii *(*)	64.29%	99.02%	52.94%	98.43%	81.65%	0.58
*Pinus thunbergii*	28.57%	99.82%	72.73%	98.61%	64.19%	0.45
*Pisum sativum*	76.92%	99.75%	74.07%	99.54%	88.34%	0.75
*Saccharum officinarum*	100.00%	99.91%	88.46%	99.91%	99.95%	0.94
*Zea mays*	96.00%	99.97%	96.00%	99.94%	97.98%	0.96
Over All	80.88%	99.81%	82.17%	99.61%	90.34%	0.81

Sen means sensitivity, Spe means specificity, PPV means positive predictive value, ACC means accuracy, BA means balanced accuracy and MCC means Matthew's correlation coefficient. All the values were obtained with leave-one-species-out cross-validation on the training set. The performance marked with "(*)" was obtained using the extended EPES definition. The overall performance was calculated using the "(*)" performance.

The extended EPES definition was applied for gymnosperm plants. With the extended EPES definition, the sensitivity on *Pinus thunbergii *was more than twice that of the original definition, the PPV only decreased by about 20%. The extended EPES definition successfully improved the performance on gymnosperms. All the following tests on gymnosperms were carried out using the extended EPES definition.

The pioneer research on predicting the C-to-U RNA editing sites in mitochondria was carried out on the balanced dataset [29]. We also built a similar balanced dataset to test the performance of our algorithm. We randomly selected a set of negatives with an equal number of positives. This set of negatives and all the positives composed the balanced dataset. The performance on this balanced dataset was almost unchanged (Table 3). The accuracy of this balanced dataset was found to be similar to the balanced accuracy of an unbalanced dataset, indicating the accuracy of a balanced dataset can be estimated by the balanced accuracy of an unbalanced dataset.

**Table 3:** The performance evaluated on a balanced dataset

Organism	Sen	Spe	PPV	ACC	BA	MCC
*Arabidopsis thaliana*	71.43%	100.00%	100.00%	85.71%	85.71%	0.75
*Atropa belladonna*	92.59%	100.00%	100.00%	96.30%	96.30%	0.93
*Nicotiana sylvestris*	91.43%	100.00%	100.00%	95.71%	95.71%	0.92
*Nicotiana tabacum*	90.63%	100.00%	100.00%	95.31%	95.31%	0.91
*Nicotiana tomentosiformis*	90.91%	100.00%	100.00%	95.45%	95.45%	0.91
*Oryza sativa*	100.00%	100.00%	100.00%	100.00%	100.00%	1.00
*Phalaenopsis aphrodite*	40.48%	100.00%	100.00%	70.24%	70.24%	0.50
*Pinus thunbergii*	64.29%	100.00%	100.00%	82.14%	82.14%	0.69
*Pisum sativum*	76.92%	100.00%	100.00%	88.46%	88.46%	0.79
*Saccharum officinarum*	100.00%	100.00%	100.00%	100.00%	100.00%	1.00
*Zea mays*	96.00%	100.00%	100.00%	98.00%	98.00%	0.96
Over All	80.88%	100.00%	100.00%	90.44%	90.44%	0.82

Sen means sensitivity, Spe means specificity, PPV means positive predictive value, ACC means accuracy, BA means balanced accuracy and MCC means Matthew's correlation coefficient. On the balanced dataset, the BA always equals the ACC.

To further eliminate the concerns of over-fitting algorithm, we carried out an independent data test. We randomly selected 10%, 20% and 30% of the dataset as the test samples. The remaining data were used as the training set. The performance estimated with this independent test was similar to the leave-one-species-out cross-validation performance (Table 4), indicating the performance of the algorithm was not over-estimated.

**Table 4:** The performance in independent tests

Test data	Sen	Spe	PPV	ACC	BA	MCC
10%	86.67%	99.74%	68.42%	99.66%	93.20%	0.77
20%	88.57%	99.68%	72.09%	99.58%	94.13%	0.80
30%	79.83%	99.75%	77.50%	99.54%	89.79%	0.78

Sen means sensitivity, Spe means specificity, PPV means positive predictive value, ACC means accuracy, BA means balanced accuracy and MCC means Matthew's correlation coefficient. The Test data column is the percentage of data that has been randomly selected as the test set. The remaining data are used as the training set.

It should be noted that the RNA editing sites of the chloroplast genes of two parasitic flowering plants, *Cuscuta reflexa *and *Cuscuta gronovii*, were recently determined [43]. These data were not deposited in the public databases and were not considered when we were developing CURE-Chloroplast. CURE-Chloroplast identified all 15 known editing sites with only two false positives in *Cuscuta reflexa *and three of the four known editing sites with only three false positives in *Cuscuta gronovii*. The overall performance in this full-blind validation achieved 94.7% sensitivity, 99.8% specificity, 78.3% PPV and 99.7% accuracy.

The parameters of CURE-Chloroplast can be adjusted in the same manner as for CURE [33]. We set the default parameters of CURE-Chloroplast to the same values as CURE. These parameters worked well enough throughout our tests. In addition, we calculated the performance of CURE-Chloroplast on *Arabidopsis thaliana *with different parameters and illustrated an ROC-like curve (Additional file 3) that describes the performance under different parameter conditions.

### Performance on entire chloroplast genome sequences

As we have described in the Implementation section, CURE-Chloroplast can directly process the entire genome sequence. Four organisms, including *Arabidopsis thaliana*, *Nicotiana tabacum*, *Zea mays *and *Pinus thunbergii*, were chosen to test the performance of CURE-Chloroplast under this condition. These organisms were chosen because they are representative of their lineages. When an organism was used as the testing sample, all the information relating to this organism was removed from the training set. The entire chloroplast genome sequence of that organism was used as the testing sequence. The results are shown in Table 5. The overall sensitivity is still over 70% under this condition. The PPV decreased because it is difficult to prevent the increment of the number of false positives when the number of negatives for testing is over 100,000 and the number of positives for testing is only about 100. The performance under this condition can be considered as a good estimation of the performance that a real user may experience, as this test condition simulates the practical application of CURE-Chloroplast.

**Table 5:** Performance test with the entire genome sequence

Organism	Sen	Spe	PPV	ACC	BA	MCC
*Arabidopsis thaliana*	67.86%	99.93%	48.72%	99.90%	83.89%	0.57
*Nicotiana tabacum*	87.50%	99.95%	65.12%	99.94%	93.72%	0.75
*Pinus thunbergii*	50.00%	99.23%	7.29%	99.17%	74.61%	0.19
*Zea Mays*	84.00%	99.94%	58.33%	99.93%	91.97%	0.70
*Over all*	72.57%	99.79%	26.45%	99.76%	86.18%	0.44

Sen means sensitivity, Spe means specificity, PPV means positive predictive value, ACC means accuracy, BA means balanced accuracy and MCC means Matthew's correlation coefficient.

### Cross-prediction between CURE and CURE-Chloroplast

The *in vivo *site recognition mechanism of the C-to-U RNA editing sites is still not understood. One hypothesis is that every single editing site or small set of editing sites is recognized by a particular factor [22,28]. The sequence patterns of the chloroplast C-to-U RNA editing sites are not expected to be shared with the mitochondria editing sites. Thus, the model trained from mitochondria and chloroplasts should not work for each other. Since we have developed CURE for predicting mitochondrial editing sites and CURE-Chloroplast for predicting chloroplast editing sites, we can see what will happen if we use the model trained on mitochondria and chloroplasts to predict the editing sites of each other.

We took *Arabidopsis thaliana *as an example. We use CURE to predict the editing sites on all edited genes in the chloroplasts and CURE-Chloroplast to predict the editing sites on all edited genes in the mitochondria. Both tests returned nothing. The model trained on chloroplasts and mitochondria cannot be used to predict the editing sites of each other. These results agree with the one-site-one-factor hypothesis.

### Can CURE-Chloroplast work on non-seed plants?

C-to-U RNA editing prediction in non-seed plants is a special problem for chloroplasts. All prediction algorithms for plant mitochondria were developed and tested with the editing sites of seed plants. Although C-to-U RNA editing sites exist in the mitochondria of non-seed plants, no computational prediction algorithm takes them into consideration.

The situation of chloroplast C-to-U RNA editing is a bit different. There are over 300 editing sites in the fern *Adiantum capillus-veneris *[44] and over 500 editing sites in the hornwort *Anthoceros formosae *[45]. The editing patterns of these two organisms are significantly different from each other and significantly different from those of seed plants. Most of the C-to-U RNA editing sites in these two species are not conserved in seed plants [44]. With the extended EPES definition, CURE-Chloroplast can achieve sensitivity 39% for *Adiantum capillus-veneris *and 51% for *Anthoceros formosae*, specificity 88% for *Adiantum capillus-veneris *and 86% for *Anthoceros formosae*, PPV 6% for *Adiantum capillus-veneris *and 14% for *Anthoceros formosae *and accuracy 87% for *Adiantum capillus-veneris *and 85% for *Anthoceros formosae*. The performance is acceptable, but lower than the performance in seed plants.

It has been suggested that the editing sites in these organisms and the seed plants are of monophyletic origin [46]. Thus, the CURE-Chloroplast algorithm should work well on these organisms. However, there are other reasons preventing CURE-Chloroplast from working well on these organisms. Technically, CURE-Chloroplast relies on accurate sequence alignment while mapping the EPESs on the target sequence. The sequence divergence between the homologous genes of these organisms and the seed plants makes it very difficult to map the EPES trained from the seed plants to the sequence of these two far related organisms.

**Figure 1:**
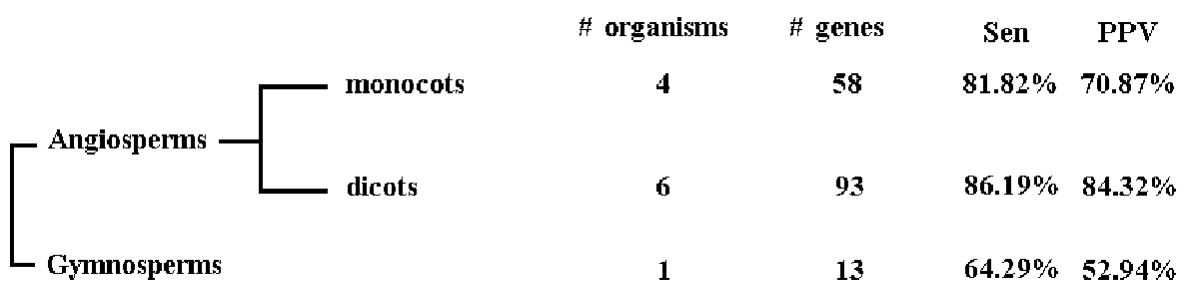
**Phylogenetically skewed knowledge of chloroplast C-to-U RNA editing sites**. Current knowledge of chloroplast C-to-U RNA editing sites is phylogenetically skewed. The performance of CURE-Chloroplast on different lineages of seed plants is associated with the abundance of data relating to that lineage. The column "# organisms" refers to the number of organisms in the corresponding lineage. The column "# genes" refers to the total number of edited genes.

Another more telling explanation for the low performance of CURE-Chloroplast with regard to these two organisms is the phylogenetically skewed knowledge of C-to-U RNA editing in chloroplasts. The performance of a lineage is associated with the abundance of data, and especially the number of organisms in that lineage with systematically determined editing sites (Figure 1). The performance for angiosperms is better than that for gymnosperms, as angiosperm data is much more comprehensive for more organisms. Although the performance for gymnosperms is also very good, it is not as good as that for angiosperms, as the data for gymnosperms is not as abundant as the data for angiosperms. Because these two organisms (*Adiantum capillus-veneris *and *Anthoceros formosae*) are the only organism with comprehensive C-to-U RNA editing information in the corresponding lineage, the low performance is expected. When the editing sites of more organisms in these lineages are determined, the performance of CURE-Chloroplast is expected to improve. However, we have to emphasize that CURE-Chloroplast is currently only developed for seed plants.

## Conclusion

CURE-Chloroplast predicts C-to-U RNA editing sites in the chloroplasts of seed plants with quite well performance. The predictive result is expected to improve as more data becomes available. The online service allows the processing of the entire chloroplast genome sequence. Although the predictive ability of CURE-Chloroplast is currently restricted within the seed plant lineage, CURE-Chloroplast is still a useful tool for studying C-to-U RNA editing in chloroplasts.

## Availability and requirements

Project name: CURE-Chloroplast

Project home page: 

Operating system(s): Online service, platform independent

Programming languages: Java, PHP, JavaScript

Other requirements: The web browser must support JavaScript

License: Free

Restrictions for non-academic use: Please contact the authors before non-academic application

## Authors' contributions

PD designed the algorithm, carried out the programming, implemented the online service, analyzed the results and partially wrote the manuscript. LJ collected the data, prepared the dataset, carried out the evaluation and optimization of the system, analyzed the results and partially wrote the manuscript. YL directed the entire study, analyzed the results and partially wrote the manuscript.

## Supplementary Material

Additional file 1 **RNAE performance on seed plants**. The prediction performance comparison between CURE-Chloroplast and RNAE. Click here for file

Additional file 2 **CURE algorithm document**. The description of CURE algorithm in details. Click here for file

Additional file 3 **ROC-like curve on *Arabidopsis thaliana***. The analysis of CURE-Chloroplast prediction performance under different algorithm parameters. Click here for file
