# Supplementary material for: CURE-Chloroplast: A chloroplast C-to-U RNA editing predictor for seed plants
Source: BMC Bioinformatics. 2009 May 8;10:135. doi: 10.1186/1471-2105-10-135 (PMC2688514; doi:10.1186/1471-2105-10-135)
Supplement: Additional file 1 — RNAE performance on seed plants. The prediction performance comparison between CURE-Chloroplast and RNAE. [file 1471-2105-10-135-S1.pdf]

Table S1 – RNAE performance on seed plants

Organism	RNAE						CURE-Chloroplast					
	**Sen**	**Spe**	**PPV**	**ACC**	**BA**	**MCC**	**Sen**	**Spe**	**PPV**	**ACC**	**BA**	**MCC**
Arabidopsis thaliana	14.29%	96.45%	4.76%	95.45%	55.37%	0.06	71.43%	99.87%	86.96%	99.52%	85.65%	0.79
Nicotiana tabacum	12.50%	91.90%	2.94%	90.59%	52.20%	0.02	90.63%	99.84%	82.86%	99.76%	95.23%	0.87
Pinus thunbergii	0.00%	96.88%	0.00%	96.14%	48.44%	-0.02	64.29%	99.02%	52.94%	98.43%	81.65%	0.58
Zea mays	12.00%	96.49%	2.27%	95.74%	54.25%	0.04	96.00%	99.97%	96.00%	99.94%	97.98%	0.96

Yura et. al. proposed a method (RNAE) for predicting C-to-U RNA editing sites in the chloroplast of *Takakia lepidozioides*. Their method seems only to work for that particular moss organism. The Sen means sensitivity. The Spe means Specificity. PPV means positive predictive value. ACC means accuracy. BA means balanced accuracy. MCC means Matthew's correlation coefficient. The performance values of RNAE are estimated with the service at http://cib.cf.ocha.ac.jp/~yura/RNAE/.
