# Supplementary material for: CURE-Chloroplast: A chloroplast C-to-U RNA editing predictor for seed plants
Source: BMC Bioinformatics. 2009 May 8;10:135. doi: 10.1186/1471-2105-10-135 (PMC2688514; doi:10.1186/1471-2105-10-135)
Supplement: Additional file 2 — CURE algorithm document. The description of CURE algorithm in details. [file 1471-2105-10-135-S2.pdf]

CURE Algorithm Document

Pufeng Du, Liyan Jia and Yanda Li*

MOE Key Laboratory of Bioinformatics and Bioinformatics Div. TNLIST /

Department of Automation, Tsinghua University, Beijing 100084, China

Supplementary material of

CURE-Chloroplast: A chloroplast C-to-U RNA editing predictor for seed plants

* Correspondence should be addressed to Yanda Li: daulyd@tsinghua.edu.cn Tel/Fax: 62794295-813

The details of CURE algorithm can be found in the following literature:

> Pufeng Du and Yanda Li: **Prediction of C-to-U RNA editing sites in plant mitochondria using both biochemical and evolutionary information**. *Journal of Theoretical Biology* 253(2008) 579-586.

For the readers' convenience, we provide a detailed description of the CURE algorithm here.

The CURE algorithm

The CURE algorithm was trained with the multi-sequences alignments of homologous genes of different organisms. The "correction" nature of C-to-U RNA editing sites implies that if one editing site is found in a column of the alignments, it is likely to find another in the same column.

The first step to predicting C-to-U RNA editing sites is to localize such columns in the input sequence. After localizing such columns, the flanking sequences which represent the biochemical important sequence features should be analyzed to improve prediction accuracy. To make describing the algorithm more convenient for us, we termed the columns containing C-to-U RNA editing sites in the alignments *Evolutionary Potential Editing Sites* (EPES). The training procedure of CURE was called EPES collection. The prediction procedure of CURE was called EPES remapping. The flowchart of the whole algorithm is shown in Figure S2.

The EPES collection procedure was carried out on the training sequence alignments. After all EPES were extracted from the sequence alignments, three descriptors including EPES consensus sequence, RNA Editing Conservative ratio and EPES micro-lib were generated for each EPES and saved in a database. EPES

consensus sequence is the consensus sequence flanking an EPES in the range -20nt to +20nt (EPES is at position 0). This range was suggested by several experiments which were dedicated to find the critical elements on the flanking sequences of editing sites [1-3]. In case the length of alignments was not sufficient to support the range -20nt to +20nt, the maximum possible length was adopted. RNA Editing Conservative ratio (REC ratio), which can be treated as the prior probability that an EPES appears to be an actual editing site in a specified species, is defined in Eqs (1). EPES micro-lib contains all sequence segments that generate the EPES consensus sequence.

$$REC\_ratio = \frac{The\ number\ of\ species\ with\ T\ on\ RNA}{The\ number\ of\ species\ with\ T\ or\ C\ on\ RNA} \qquad (1)$$

The EPES remapping procedure was mainly based on the BLAST search of EPES consensus sequence in the input sequence. Every EPES consensus sequence was used as a query sequence of BLAST to find the High Score Pair (HSP) in the input sequence. After the input sequence was aligned by a set of EPES consensus sequences, every aligning EPES was examined. If the REC ratio of the aligning EPES was not in the micro-lib classifier working region which was bounded by the upper cutoff and the lower cutoff, the prediction result was determined directly by the REC ratio. If the REC ratio cannot directly determine the prediction result, the K Nearest Neighbor (KNN) classifier which was trained on the micro-lib was used to make the final prediction. The input sequence segment which was aligned by the EPES consensus sequence was classified by this KNN classifier. The prediction result was generated according to the result of the KNN classifier. Hamming distance was adopted when calculating the distance between sequences. According to our

experience, K = 1 is enough to get an optimal result.

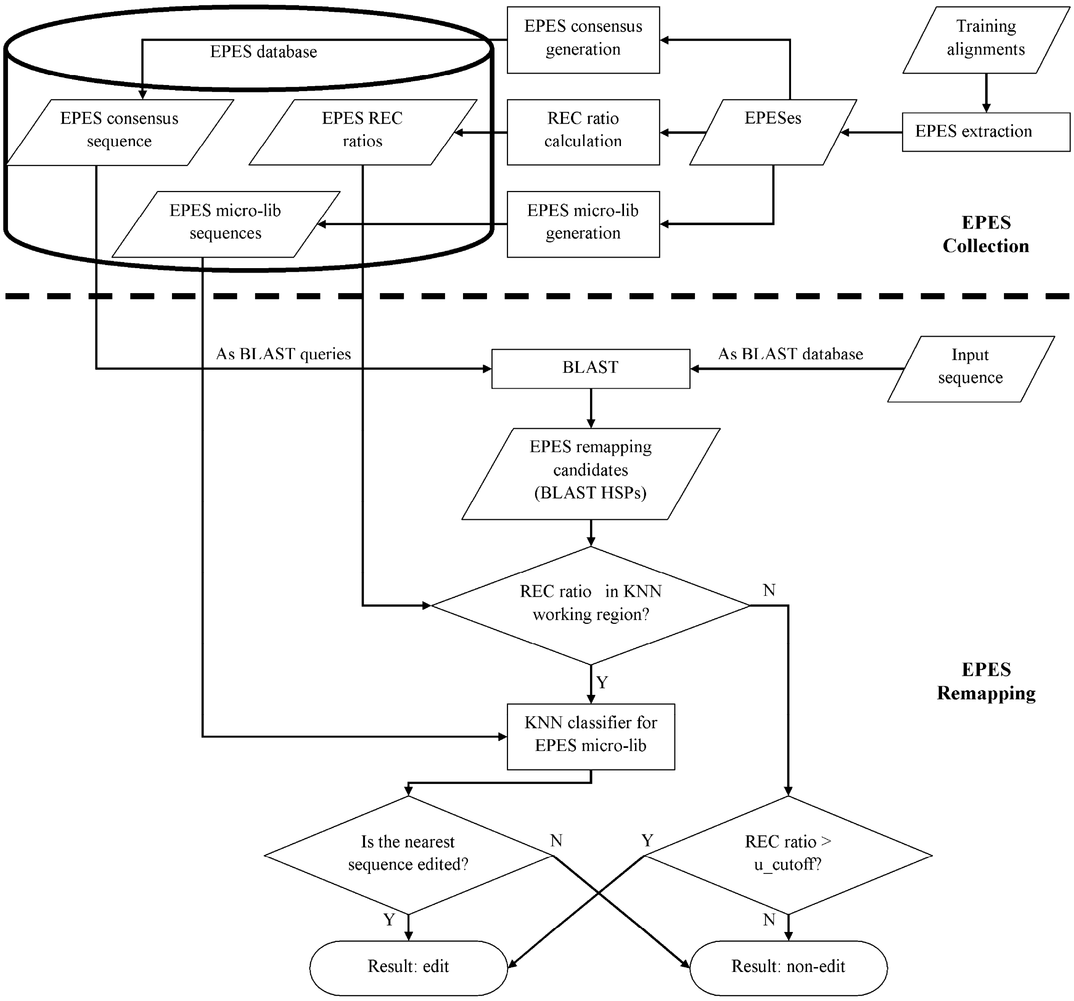

Figure S2 - The flowchart of the CURE algorithm. The training alignments is the dataset prepared in section Dataset preparation. The “u_cutoff” is the upper bound of the KNN working region.

References

1. Hayes ML, Reed ML, Hegeman CE, Hanson MR: **Sequence elements critical for efficient RNA editing of a tobacco chloroplast transcript in vivo and in vitro**. *Nucleic Acids Res* 2006, **34**:3742-3754.
2. Takenaka M, Neuwirt J, Brennicke A: **Complex cis-elements determine an RNA editing site in pea mitochondria**. *Nucleic Acids Res* 2004, **32**:4137-4144.
3. Bock R, Hermann M, Fuchs M: **Identification of critical nucleotide positions for plastid RNA editing site recognition**. *RNA* 1997, **3**:1194-1200.
