# Supplementary material for: CURE-Chloroplast: A chloroplast C-to-U RNA editing predictor for seed plants
Source: BMC Bioinformatics. 2009 May 8;10:135. doi: 10.1186/1471-2105-10-135 (PMC2688514; doi:10.1186/1471-2105-10-135)
Supplement: Additional file 3 — ROC-like curve on Arabidopsis thaliana. The analysis of CURE-Chloroplast prediction performance under different algorithm parameters. [file 1471-2105-10-135-S3.pdf]

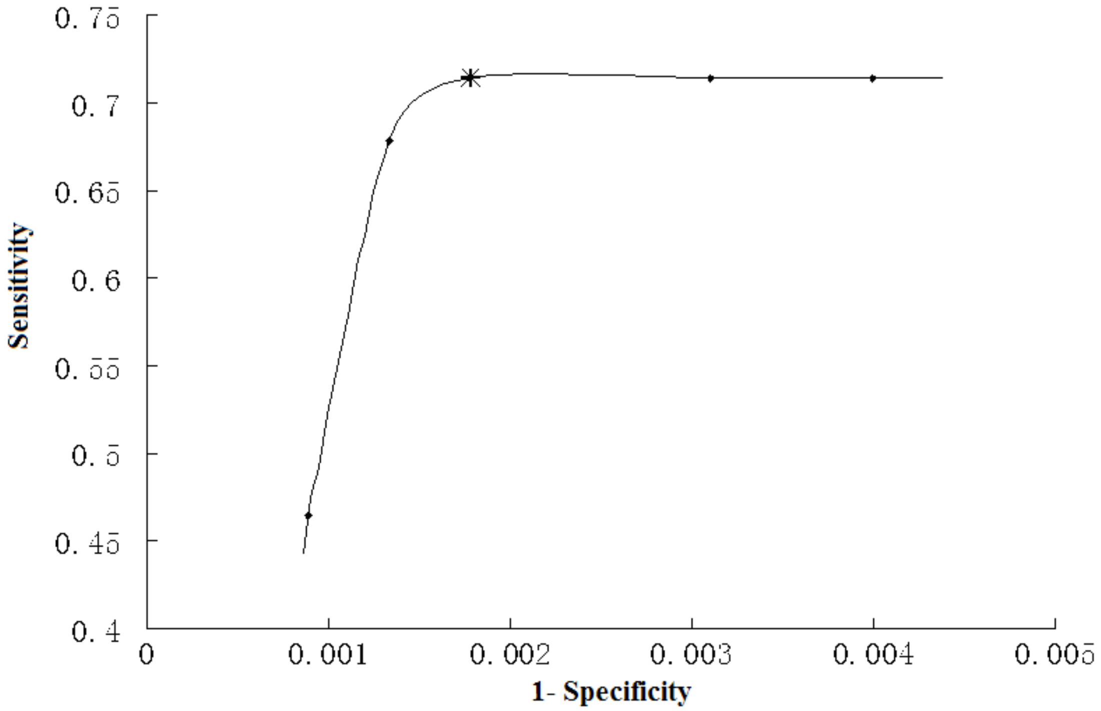

Figure S1 – The ROC-like (Receiver Operating Characteristic) curve of CURE-Chloroplast. The sensitivity and specificity changed with different upper bound and lower bound parameters. The X axis is the 1-specificity. The Y axis is the sensitivity. The default parameter of CURE-Chloroplast is marked with “*”.
